# KBTBD11, a novel BTB-Kelch protein, is a negative regulator of osteoclastogenesis through controlling Cullin3-mediated ubiquitination of NFATc1

**DOI:** 10.1038/s41598-019-40240-2

**Published:** 2019-03-05

**Authors:** Shun Narahara, Eiko Sakai, Tomoko Kadowaki, Yu Yamaguchi, Haruna Narahara, Kuniaki Okamoto, Izumi Asahina, Takayuki Tsukuba

**Affiliations:** 1 Department of Dental Pharmacology, Graduate School of Biomedical Sciences, Nagasaki University, Nagasaki, 852-8588 Japan; 2 Department of Regenerative Oral Surgery, Graduate School of Biomedical Sciences, Nagasaki University, Nagasaki, 852-8588 Japan; 3 Department of Frontier Life Science, Graduate School of Biomedical Sciences, Nagasaki University, Nagasaki, 852-8588 Japan; 4 Department of Dental Pharmacology, Okayama University Graduate School of Medicine, Dentistry and Pharmaceutical Sciences, Okayama, 700-8525 Japan

## Abstract

Kelch repeat and BTB domain-containing protein 11 (KBTBD11) is a member of the KBTBD subfamily of proteins that possess a BTB domain and Kelch repeats. Despite the presence of the *Kbtbd11* gene in mammalian genomes, there are few reports about KBTBD11 at present. In this study, we identified the novel protein KBTBD11 as a negative regulator of osteoclast differentiation. We found that expression of KBTBD11 increased during osteoclastogenesis. Small-interfering-RNA-mediated knockdown of KBTBD11 enhanced osteoclast formation, and markedly increased the expression of several osteoclast marker genes compared with control cells. Conversely, KBTBD11 overexpression impaired osteoclast differentiation, and decreased the expression of osteoclast marker genes. Among six major signaling pathways regulating osteoclast differentiation, KBTBD11 predominantly influenced the nuclear factor of activated T cell cytoplasmic-1 (NFATc1) pathway. Mechanistically, KBTBD11 was found to interact with an E3 ubiquitin ligase, Cullin3. Further experiments involving immunoprecipitation and treatment with MG132, a proteasome inhibitor, showed that the KBTBD11–Cullin3 promotes ubiquitination and degradation of NFATc1 by the proteasome. Considering that NFATc1 is an essential factor for osteoclast differentiation, the KBTBD11 and Cullin3 probably regulate the levels of NFATc1 through the ubiquitin-proteasome degradation system. Thus, KBTBD11 negatively modulates osteoclast differentiation by controlling Cullin3-mediated ubiquitination of NFATc1.

## Introduction

Osteoclasts are multinucleated giant cells mainly responsible for bone resorption^1,2^. Osteoclasts are formed by the fusion of mononuclear monocyte/macrophage progenitor cells. Osteoclast differentiation is regulated by the essential cytokines: receptor activator of nuclear factor κB ligand (RANKL) and macrophage colony-stimulating factor (M-CSF). Interaction between RANKL and its receptor (RANK) triggers the major differentiation-related signaling pathways, such as the signaling through nuclear factor of activated T cells cytoplasmic-1 (NFATc1), the signaling via p38 mitogen-activated protein kinase (MAPK), the signaling cascade involving extracellular signal-regulated kinase (ERK), the signaling through Jun N-terminal kinase (JNK), the signal transduction via phosphatidylinositol 3-kinase (PI3K)/Akt, and the signaling mediated by nuclear factor kappa B (NF-κB)^3–6^. In addition to signaling mechanisms, recent studies have revealed the importance of epigenetic mechanisms in the regulation of osteoclast differentiation, including post-translational modifications of DNA and proteins as well as expression of noncoding RNA^7,8^. In particular, ubiquitination and subsequent proteasomal degradation have been reported to be involved in the regulation of osteoclastogenesis^9,10^. It is generally accepted that various ubiquitin ligases regulate the protein level of signaling molecules via proteasome-dependent degradation^11^. For example, Cbl-b and c-Cbl, the RING finger-type E3 ubiquitin ligases, control osteoclast differentiation through ubiquitin-mediated downregulation of Src and NFATc1^12–15^. The HECT-type Nedd4-like E3 ubiquitin ligase, Itch, is also involved in osteoclast differentiation by promoting deubiquitination of Tumor Necrosis Factor (TNF) receptor–associated factor 6 (TRAF6)^16^. Itch-deficient osteoclast precursors display extended NF-κB activation and delayed deubiquitination of TRAF6^16^. Although it is speculated that other ubiquitin ligases also regulate osteoclast differentiation, the detailed mechanisms remain completely unknown.

Recently, our research group performed DNA microarray analysis of osteoclast differentiation showing that 1,363 genes are upregulated, and 881 genes are downregulated^17^. Among the upregulated genes, we identified a novel gene, termed as Kelch repeat and BTB domain-containing protein 11 (*Kbtbd11*), which is possibly implicated in an E3 ubiquitin ligase-mediated event. Based on its genetic information, the deduced amino acid sequence of KBTBD11 indicates that this protein contains an N-terminal BTB/POZ domain and a C-terminal Kelch-repeat domain. The BTB domain is derived from a sequence homology of Drosophila Bric-a-brac, Tramtrack, and Broad complex^18^. The BTB domain participates in protein–protein interactions, including self-oligomerization and interaction with other proteins^19^. In many cases, the BTB domain acts as a specific adaptor for Cullin3, a RING-finger type E3 ubiquitin ligase that mediates ubiquitin-dependent degradation by the proteasome^20^. The Kelch-repeat domain forms β-propellers that generally take part in protein-protein interactions^21^. Nonetheless, there is few information about the function of KBTBD11 in mammalian cells at present. Therefore, the genetic information prompted us to test whether KBTBD11 is responsible for osteoclastogenesis via E3 ubiquitin ligase-mediated events.

In this study, we investigated the role of KBTBD11 in osteoclastogenesis of mouse macrophage-like RAW-D cells by gene knockdown using small interfering RNAs (siRNAs) or gene overexpression systems. Here we show that KBTBD11 negatively regulates osteoclast differentiation by interaction with Cullin3, followed by NFATc1 degradation.

## Results

### KBTBD11 is upregulated during osteoclast differentiation

**Figure 1:**
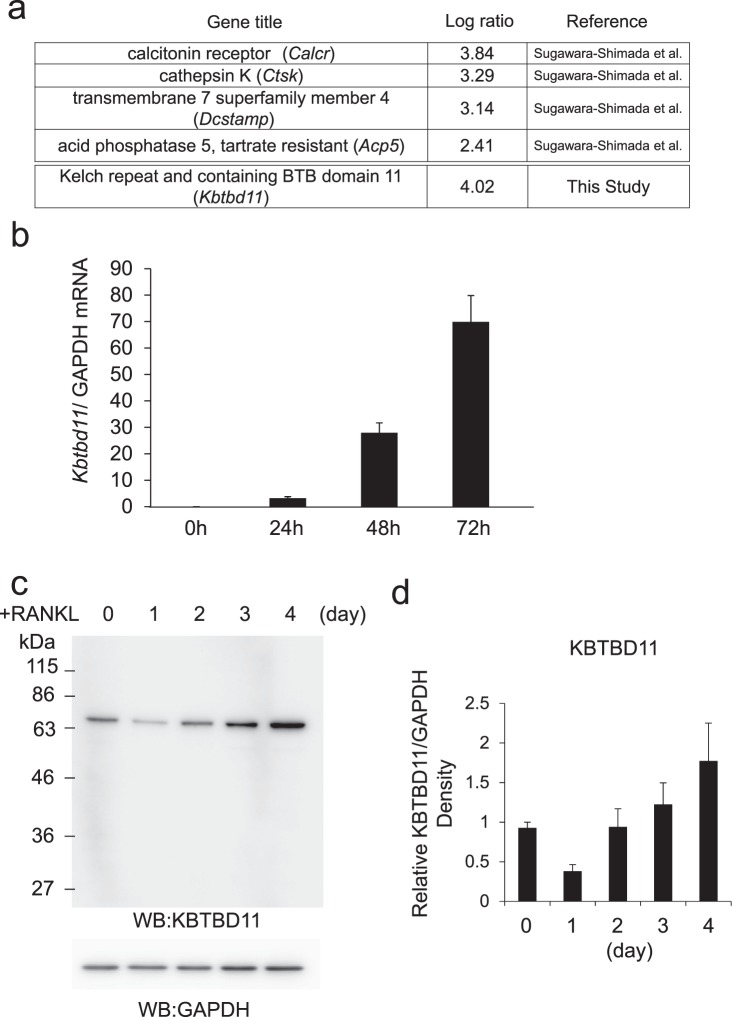
Expression levels of KBTBD11 in osteoclasts. (**a**) A list of upregulated transcripts in rapidly differentiating osteoclasts compared to slow differentiating osteoclasts. Several marker genes were described previously^17^. (**b**) The mRNA expression of KBTBD11 was measured by RT-PCR analysis during osteoclast differentiation in RAW-D cells with 100 ng/mL RANKL. The data are presented as the mean ± S.D. of values from 3 independent experiments. (**c**) The protein expression of KBTBD11 in RAW-D cells was cultured with RANKL (100 ng/mL) for 0–4 days. Cell lysates were subjected to SDS-PAGE followed by western blotting with antibodies against KBTBD11 and GAPDH. (**d**) Densitometric analysis for the quantification of each protein in the cell lysate of both cell types as shown in (**c**). The relative levels were defined as the chemiluminescence intensity per mm^2^ measured by LAS4000-mini. The data are presented as the mean ± S.D. of values from 3 independent experiments.

Recently, by DNA microarray analysis, our group demonstrated that 1,363 genes are upregulated and 881 genes are downregulated during osteoclastogenesis of bone-marrow macrophages^17^. Among the upregulated genes, we found an uncharacterized gene, *Kbtbd11*, as well as other osteoclast marker genes, such as calcitonin receptor (*Calcr*), cathepsin K (*Ctsk*), transmembrane 7 superfamily member 4 (*Dcstamp*), and tartrate-resistant acid phosphatase (*Acp5*; Fig. 1a).

To explore whether the expression level of KBTBD11 increases during osteoclast differentiation, we determined the mRNA level of *Kbtbd11* in mouse macrophage-like RAW-D cells. Determination by real-time polymerase chain reaction (RT-PCR) showed that *Kbtbd11* level gradually increased after RANKL stimulation (Fig. 1b). The mRNA level of *Kbtbd11* at 72 h after stimulation reached a ~70-fold higher level than that in unstimulated cells (Fig. 1b). We also examined the protein levels of KBTBD11 in RAW-D cells during RANKL-induced osteoclastogenesis (Fig. 1c). Western blot analysis revealed that the endogenous KBTBD11 in RAW-D cells was detected as a protein with a molecular mass of ~67 kDa. The KBTBD11 levels in RANKL-stimulated cells gradually increased as compared with unstimulated cells, although this level transiently decreased after 1 day of stimulation (Fig. 1c). Thus, KBTBD11 was upregulated during osteoclast differentiation.

### Knockdown of KBTBD11 enhances osteoclast differentiation

**Figure 2:**
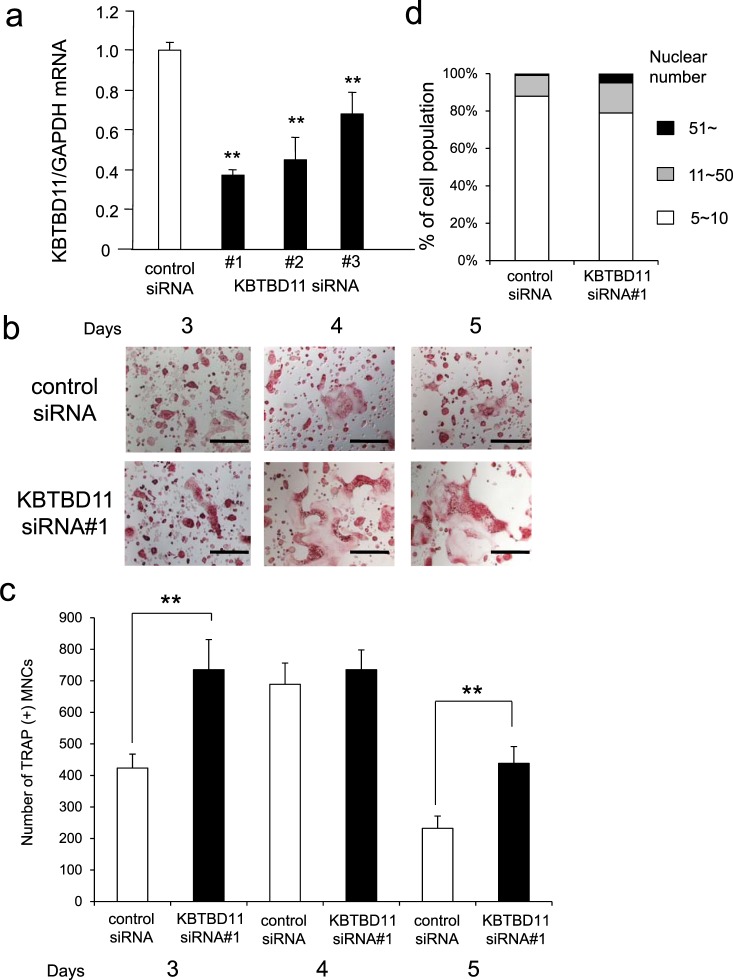
Knockdown of KBTBD11 in osteoclasts. (**a**) The efficacy of KBTBD11 knockdown was evaluated by measuring its mRNA levels. Cells were transfected with control or KBTBD11-specific siRNA (10 pmol) for 24 h, and subsequently incubated with RANKL (100 ng/mL) for additional 24 h. The data are presented as the mean ± S.D. of values from 3 independent experiments. **P < 0.01 for the indicated comparison. (**b**) TRAP staining of control and KBTBD11-knockdown osteoclasts. The control and KBTBD11-depleted RAW-D cells were stimulated with RANKL (100 ng/mL) for 4 days. The cells were fixed and stained with TRAP. Scale bars: 200 μm. (**c**) The numbers of TRAP-positive multinuclear cells (MNCs) in control and KBTBD11 knockdown cells were determined on the indicated days. The data are presented as the mean ± S.D. of values from 3 independent experiments. **P < 0.01 for the indicated comparison. (**d**) The nuclear numbers of control and KBTBD11 knockdown cells were counted and classified as 5–10 (white bar), 11–50 (gray bars) or more than 51 (black bar).

To study the role of KBTBD11 during osteoclast differentiation, we performed siRNA-mediated knockdown experiments. The efficacy of the KBTBD11 knockdown in RANKL-stimulated RAW-D cells was determined (Fig. 2a). Depletion of KBTBD11 by siRNA #1 in the cells yielded an approximately 60% reduction, whereas siRNA #2, and #3 caused an approximately 50% and 30% reduction, respectively, as compared to the control siRNA (Fig. 2a). Therefore, we selected siRNA #1 for following knockdown experiments, because the knockdown efficacy was the highest. Upon stimulation with RANKL for 3–5 days, KBTBD11-depleted cells displayed larger formation in osteoclasts compared with the control (Fig. 2b). The number of TRAP-positive multinucleated cells (MNCs) was significantly higher in KBTBD 11 knockdown cells than that in control cells at 3 and 5 days (Fig. 2c). The number of control cells peaked on the 4th day after stimulation and fell immediately on the 5th days (Fig. 2c). In KBTBD11 knockdown cells, however, the peaks of number persisted for 3 to 4 days, and this number was declined on the 5th day of stimulation (Fig. 2c). Moreover, the nuclear number of KBTBD11-knockdown osteoclasts was greater than that of control osteoclasts (Fig. 2d).

To determine whether KBTBD11 knockdown enhances osteoclast maturation, we measured the mRNA expression of some osteoclast marker genes. RT-PCR analysis showed that the expression of *Nfatc1*, *Src*, *Ctsk*, osteoclast stimulatory transmembrane protein (*Ocstamp*), *Dcstamp*, and matrix metalloprotease 9 (*Mmp9*) was significantly higher in KBTBD11-knockdown osteoclasts than in control cells (Supplementary Fig. S1A). These results imply that KBTBD11-depleted osteoclasts display enhanced maturation compared with control cells.

**Figure 3:**
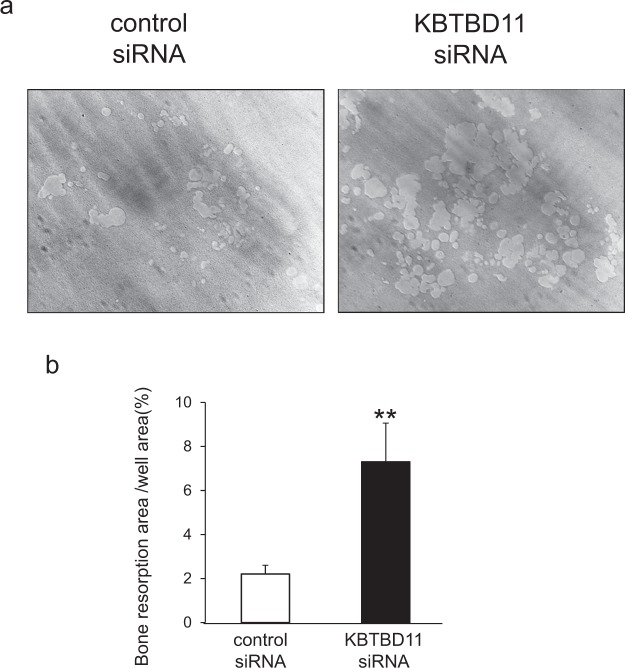
Bone-resorbing activities of control and KBTBD11-knockdown osteoclasts. (**a**) Control and KBTBD11-knockdown RAW-D cells were seeded on Osteo Assay Stripwell Plates with RANKL (500 ng/mL) and incubated for 7 days. Photographs of the bone-resorbing activity of each osteoclast. (**b**) The resorption area was determined using Image J software. Data are presented as the mean ± S.D. of 3 independent experiments. **P < 0.01 for the indicated comparisons.

Next, to investigate bone resorption activity, we performed a pit formation assay on control and KBTBD11-depleted osteoclasts. As expected, KBTBD11-knockdown osteoclasts showed a marked increase in resorption activity compared with control osteoclasts (Fig. 3a). When we calculated the resorption area, this area generated by KBTBD11-knockdown osteoclasts was significantly larger than that generated by the control osteoclasts (Fig. 3b).

**Figure 4:**
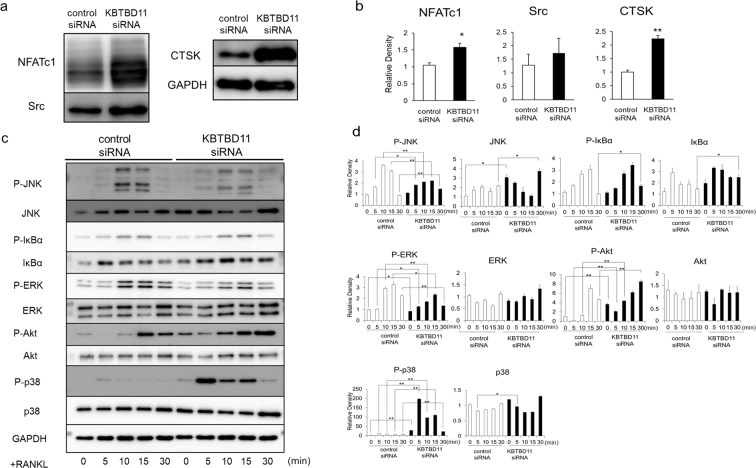
Signaling levels of control or KBTBD11-knockdown RAW-D cells stimulated with RANKL. (**a**) Control or KBTBD11-knockdown RAW-D cells were cultured with RANKL (100 ng/mL) for 1 day. Cell lysates (same protein amounts) were subjected to SDS-PAGE followed by western blotting with antibodies against NFATc1, Src, CTSK, and GAPDH (control). (**b**) Densitometric analysis for the quantification of each protein in the cell lysate of both cell types as shown in (**a**). The relative levels were defined as the chemiluminescence intensity per mm^2^ measured by LAS4000-mini. The data are presented as the mean ± S.D. of values from 3 independent experiments. *P < 0.05, **P < 0.01 for the indicated comparison. (**c**) Control or KBTBD11-knockdown RAW-D cells were preincubated for 2 h in the serum-free media in the absence of RANKL. After addition of RANKL, the cells were incubated for the indicated time periods, consequently harvested. Cell lysates (same protein amounts) were subjected to SDS-PAGE followed by western blotting with antibodies against p-JNK, p-IκBα, p-ERK, p-Akt, p-p38, JNK, IκBα, ERK, Akt, p38, and GAPDH (control). (**d**) Densitometric analysis for the quantification of each protein in the cell lysate of both cell types as shown in (**c**). The relative levels were defined as the chemiluminescence intensity per mm^2^ measured by LAS4000-mini. The data are presented as the mean ± S.D. of values from 3 independent experiments. *P < 0.05, **P < 0.01 for the indicated comparison.

To explore the molecular mechanisms underlying the enhanced maturation of KBTBD11-knockdown osteoclasts, we investigated the expression of signaling molecules in control and KBTBD11-knockdown RAW-D macrophages after RANKL stimulation. Western blot analysis revealed that the protein level of NFATc1 and CTSK in KBTBD11-knockdown RAW-D cells was markedly higher than that in control cells (Fig. 4a,b). Moreover, the protein levels of Src were slightly higher in KBTBD11-knockdown RAW-D cells than in control cells, although there was no statistically significant difference (Fig. 4a,b). Upon stimulation with RANKL, the phosphorylation levels of p-38 and Akt increased in KBTBD11-knockdown RAW-D cells compared to control cells (Fig. 4c,d). However, the phosphorylation levels of IκB were comparable between control and KBTBD11-knockdown RAW-D cells (Fig. 4c,d). Conversely, the phosphorylation levels of JNK and ERK were lower in KBTBD11-knockdown RAW-D cells than in control cells (Fig. 4c,d).

### Overexpression of KBTBD11 impairs osteoclast differentiation

**Figure 5:**
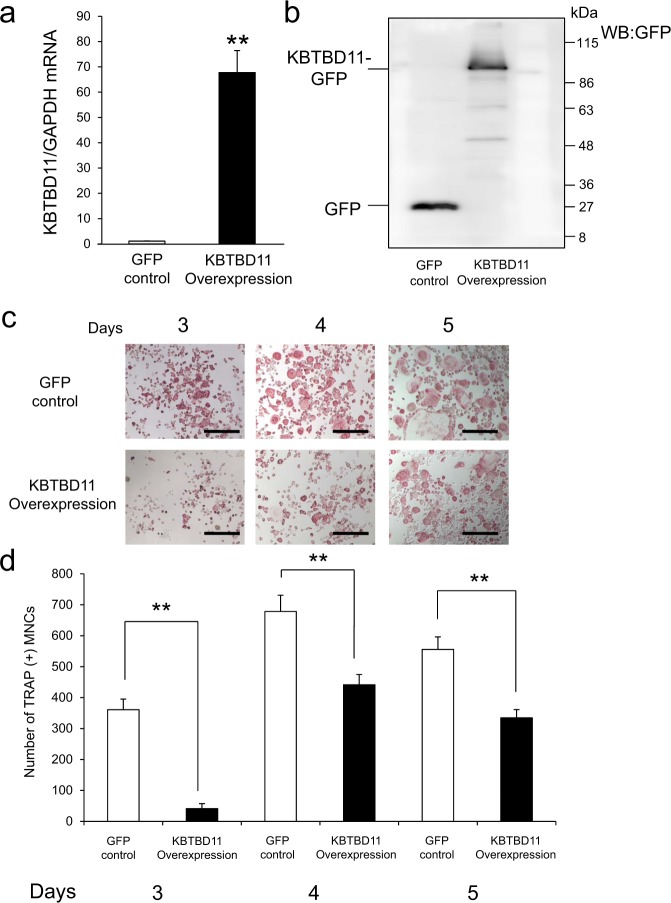
Overexpression of KBTBD11 in osteoclasts. RAW-D cells were transduced with a vector containing either GFP-tagged KBTBD11 or only EGFP (control). (**a**) Quantitative RT-PCR analysis of KBTBD11 mRNA expression in RAW-D cells expressing GFP or GFP-KBTBD11. The data are represented as mean ± SD of values from five independent experiments. **P < *0.01*; compared with the control cells. (**b**) The cultured cells were harvested on day 3, and cell lysates from control and KBTBD11-overexpressing RAW-D cells were subjected to SDS-PAGE, followed by western blotting with an anti-GFP antibody. (**c**) TRAP staining of control and KBTBD11-overexpressing osteoclasts. The control and KBTBD11-overexpressing RAW-D cells were stimulated with RANKL (100 ng/mL) for 3–5 days. The cells were fixed and stained with TRAP. Scale bars: 200 μm. (**d**) The number of TRAP-positive multinuclear cells (MNCs) in control and KBTBD11 overexpressing cells was counted on the indicated days. The data are presented as the mean ± S.D. of values from 3 independent experiments. **P < 0.01 for the indicated comparison.

To confirm that KBTBD11 overexpression prevents osteoclast differentiation, we performed overexpression experiments on RAW-D cells with a vector encoding KBTBD11-green fluorescent protein (GFP) fusion or GFP alone (control). The mRNA expression level of KBTBD11-GFP increased by ~70-fold compared with that of control cells (Fig. 5a). Western blot analysis with anti-GFP antibody revealed that the KBTBD11-GFP fusion protein was detected as a band with a molecular mass of approximately 95 kDa (Fig. 5b). Upon RANKL stimulation for 3–5 days, TRAP staining analysis indicated that KBTBD11-overexpressing osteoclasts were apparently smaller in size compared to control osteoclasts (Fig. 5c). After the number of TRAP-positive MNCs was determined, the number of KBTBD11-overexpressing osteoclasts was significantly lower than that of control osteoclasts (Fig. 5d).

To determine investigate whether KBTBD11-overexpressing osteoclasts undergo delayed maturation, we measured the mRNA levels of some osteoclast marker genes in control and KBTBD11-overexpressing osteoclasts (Supplementary Fig. S2). Control and KBTBD11-overexpressing RAW-D cells were cultured with RANKL (100 ng/mL) for 3 days. RT-PCR analysis of cells in both groups showed that mRNA levels of *Nfatc1*, *Src*, *Ctsk*, *Ocstamp*, and *Mmp9* were significantly lower in KBTBD11-overexpressing osteoclasts compared with control cells, although the mRNA levels of *Dcstamp* were comparable between control and KBTBD11-overexpressing osteoclasts (Supplementary Fig. S2).

We further compared bone resorption activities between control and KBTBD11-overexpressing osteoclasts. The pit formation assay showed that KBTBD11-overexpressing osteoclasts exhibited a markedly lower resorption activity compared with control osteoclasts (Supplementary Fig. S3A). The respective calculated resorption area of KBTBD11-overexpressing osteoclasts was significantly lower than that of control osteoclasts (Supplementary Fig. S3B). These results indicate that KBTBD11 overexpression impairs almost marker gene expression and bone resorption activity in such osteoclasts compared with control osteoclasts.

**Figure 6:**
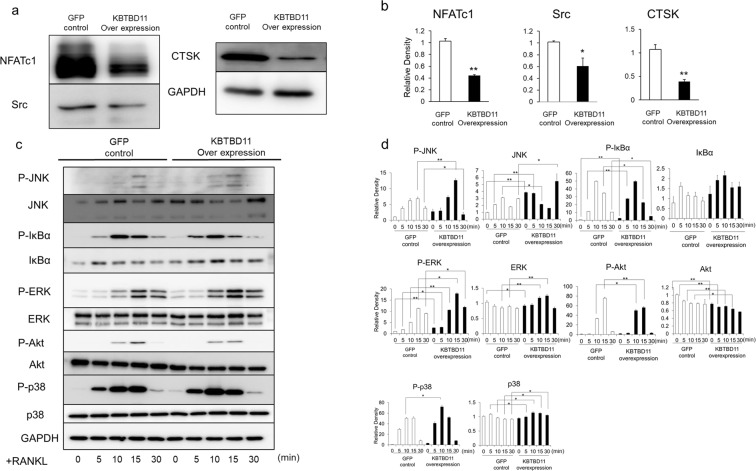
Signaling levels of control or KBTBD11-overexpressing RAW-D cells stimulated with RANKL. (**a**) Control or KBTBD11-overexpressing RAW-D cells were cultured with RANKL (100 ng/mL) for 1 day. Cell lysates (same protein amounts) were subjected to SDS-PAGE followed by western blotting with antibodies against Src, NFATc1, CTSK, and GAPDH (control). (**b**) Densitometric analysis for the quantification of each protein in the cell lysate of both cell types as shown in (**a**). The relative levels were defined as the chemiluminescence intensity per mm^2^ measured by LAS4000-mini. The data are presented as the mean ± S.D. of values from 3 independent experiments. *P < 0.05, **P < 0.01 for the indicated comparison. (**c**) Control or KBTBD11-overexpressing RAW-D cells were preincubated for 2 h in the serum-free media in the absence of RANKL. After addition of RANKL, the cells were incubated for the indicated times, consequently harvested. Cell lysates (same protein amounts) were subjected to SDS-PAGE followed by western blotting with antibodies against p-JNK, p-IκBα, p-ERK, p-Akt, p-p38, JNK, IκBα, ERK, Akt, p38, and GAPDH (control). (**d**) Densitometric analysis for the quantification of each protein in the cell lysate of both cell types as shown in (**c**). The relative levels were defined as the chemiluminescence intensity per mm^2^ measured by LAS4000-mini. The data are presented as the mean ± S.D. of values from 3 independent experiments. *P < 0.05, **P < 0.01 for the indicated comparison.

Next, to examine signaling pathways affected in KBTBD11-overexpressing osteoclasts, we analyzed the signaling molecules in control and KBTBD11-overexpressing RAW-D cells upon stimulation with RANKL (Fig. 6). Western blot analysis showed that expression levels of NFATc1 and CTSK were markedly decreased in KBTBD11-overexpressing RAW-D cells compared with control cells (Fig. 6a). In addition, the protein levels of Src were also lower in KBTBD11-overexpressing RAW-D cells than in control cells (Fig. 6a). We also examined nuclear translocation of NFATc1 in control and KBTBD11-overexpressing osteoclasts. As expected, the levels of nuclear-translocated NFATc1 in KBTBD11-overexpressing cells were significantly lower than those in control cells (Supplementary Fig. S4). Upon stimulation with RANKL, the phosphorylation levels of some relevant molecules, such as IκB and p-38 were almost indistinguishable between control and KBTBD11-overexpressing RAW-D cells, although there are some differences in each time (Fig. 6c,d). The phosphorylation levels of JNK and ERK were slightly higher in KBTBD11-overexpressing RAW-D cells than in control cells (Fig. 6c,d). However, the phosphorylation levels of Akt decreased in KBTBD11-overexpressing RAW-D cells compared to control cells (Fig. 6c,d).

### KBTBD11 interacts with the E3 ubiquitin ligase Cullin3

**Figure 7:**
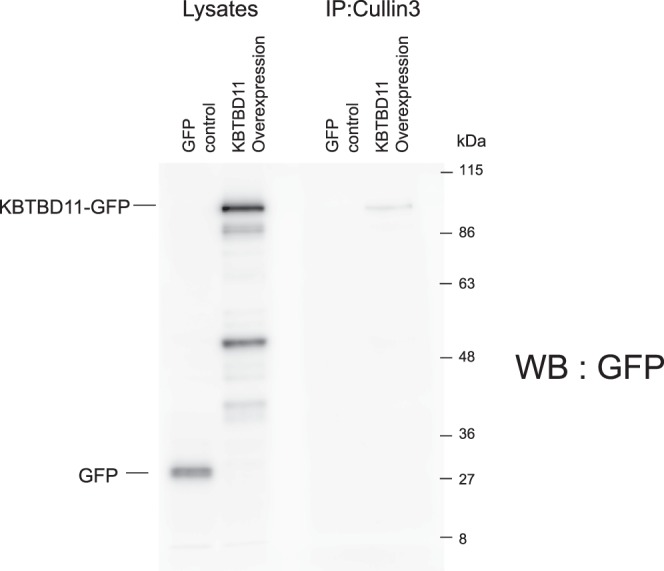
Interaction of KBTBD11 with the ubiquitin ligase Cullin3. Control or KBTBD11-overexpressing RAW-D cells were cultured with RANKL (100 ng/mL) for 2 days. The cells were lysed with PBS containing 0.1% Triton X-100. After centrifugation, the supernatants were immunoprecipitated with anti-Cullin3 antibody. After addition of protein G–Sepharose, the beads were resuspended in Laemmli’s sample buffer, and subjected to western blot analysis using anti-GFP antibody. The similar experiments were performed independently more than 4 times.

Generally, Kelch-BTB proteins function as adaptor proteins of the E3 ubiquitin ligase Cullin3^20^. Therefore, we examined whether KBTBD11 interacts with Cullin3. The cell lysates from control and KBTBD11-overexpressing RAW-D cells were immunoprecipitated with an anti-Cullin3 antibody, and the immune-complex was subjected to western blot analysis with an anti-GFP antibody. The coimmunoprecipitated protein band corresponding to KBTBD11-GFP was detected at a molecular mass of approximately 95 kDa (Fig. 7). These results suggest that KBTBD11 interacts with Cullin3.

### NFATc1 is a ubiquitinated substrate for KBTBD11 and Cullin3

**Figure 8:**
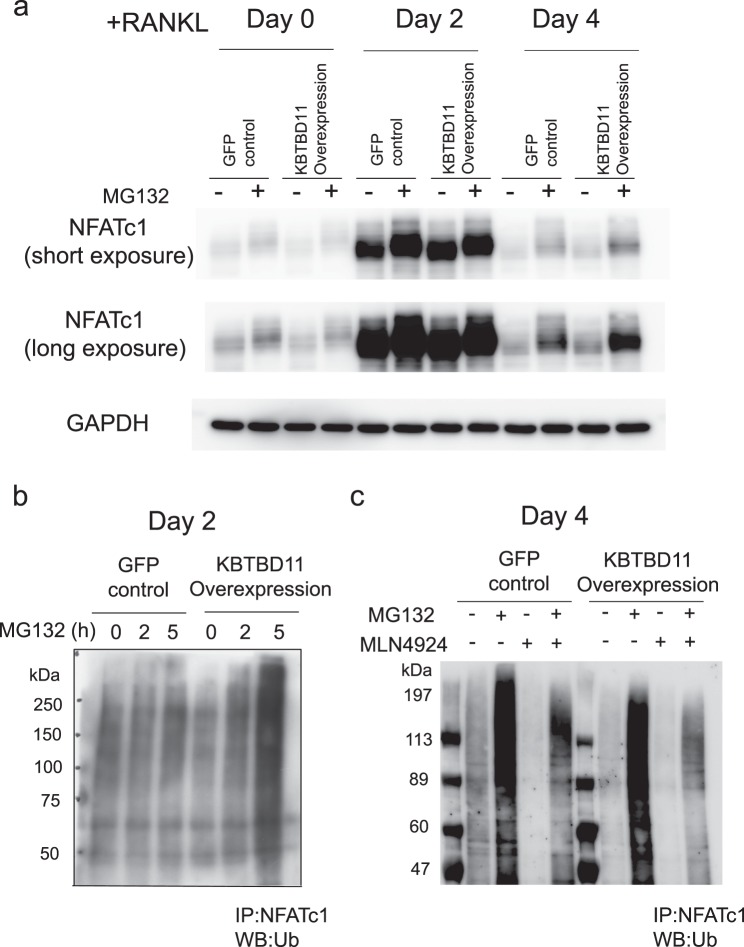
Possible involvement of KBTBD11 and Cullin3 in ubiquitination of NFATc1. (**a**) Control or KBTBD11-overexpressing RAW-D cells were cultured with RANKL (100 ng/mL) for 0–4 days. The cells were incubated without or with 20 μM MG132 for 5 h, and were lysed in a lysis buffer. The lysates were subjected to western blot analysis using anti-NFATc1 and anti-GAPDH antibodies. (**b**) Control or KBTBD11-overexpressing RAW-D cells were cultured with RANKL (100 ng/mL) for 2 days. The cells were incubated without or with 20 μM MG132 for 5 h, and were lysed in a lysis buffer. The lysates were immunoprecipitated with anti-NFATc1 anti-body. After addition of protein G–Sepharose, the binding proteins were eluted and subjected to western blot analysis using anti-ubiquitin. (**c**) Control or KBTBD11-overexpressing RAW-D cells were cultured with RANKL (100 ng/mL) for 4 days. The cells were incubated without or with 20 μM MG132 and 10 nM MLN4924 for 5 h, and were lysed in a lysis buffer. The lysates were immunoprecipitated with anti-NFATc1 anti-body. After addition of protein G–Sepharose, the binding proteins were eluted and subjected to western blot analysis using anti-ubiquitin. The experiments were performed independently 3 times.

When we analyzed some candidate proteins as substrates, we found NFATc1 to be a ubiquitinated substrate (Fig. 8). As shown in Fig. 8a, the NFATc1 expression was markedly induced on day 2 after RANKL stimulation, but it decreased on day 4. Treatment with MG132, an inhibitor of the proteasome, induced accumulation of NFATc1 on day 0 and day 4 after RANKL stimulation, although it slightly increased the levels of NFATc1 on day 2 (Fig. 8a). However, accumulation of NFATc1 on day 2 after RANKL stimulation was found as poly-ubiqutinated proteins (Fig. 8b). To analyze ubiquitinated NFATc1, we performed immunoprecipitation with anti-NFATc1 antibody followed by western blot analysis with anti-ubiquitin antibody (Fig. 8b and c). After RANKL stimulation for 2 days, the levels of ubiquitinated NFATc1 markedly increased in KBTBD11-overexpressing cells treated with MG132 for 5 h compared with untreated cells (Fig. 8b). In MG132-treated control cells, the levels of ubiquitinated NFATc1 were slightly higher than those in untreated cells (Fig. 8b). These results indicate that the presence of KBTBD11 enhances ubiquitination of NFATc1. To confirm whether the KBTBD11 and Cullin3 affects ubiquitination of NFATc1, we treated the control and KBTBD11-overexpressing cells with MLN4924, which works as an inhibitor of Cullin ligases by blocking neddylation of the enzyme (Fig. 8c). After RANKL stimulation for 4 days, immunoprecipitation for ubiquitinated NFATc1 revealed that MLN4924 treatment markedly inhibited the levels of ubiquitinated NFATc1 in MG132-treated cells, suggesting the involvement of a Cullin ligase(s) in ubiquitination of NFATc1 (Fig. 8c). Taken together, these results indicate that NFATc1 is possibly one of the substrates ubiquitinated under the influence of the KBTBD11 and Cullin3.

## Discussion

In this study, we showed that KBTBD11 expression increased during osteoclast differentiation. The KBTBD11 knockdown enhanced osteoclastogenesis; namely, KBTBD11-depleted osteoclasts displayed larger formation than control osteoclasts and showed increased expression of osteoclast marker genes. In contrast, KBTBD11 overexpression abrogated osteoclast differentiation concomitant with decreased expression of osteoclast marker genes. Among the major signaling pathways involved in osteoclast differentiation, KBTBD11 predominantly affected NFATc1 signaling. Mechanistically, KBTBD11 interacted with the E3 ubiquitin ligase Cullin3. Moreover, KBTBD11 overexpression induced accumulation of ubiquitinated NFATc1, indicating that the KBTBD11 and Cullin3 participate in degradation of ubiquitinated NFATc1 by the proteasome. Given that NFATc1 is known to regulate osteoclast differentiation, the KBTBD11 and Cullin3 are probably involved in regulating the protein levels of NFATc1 by ubiquitination. Thus, KBTBD11 negatively regulates osteoclast differentiation by means of interaction with Cullin3 via NFATc1 degradation.

Based on these findings, the KBTBD11 expression is induced during osteoclastogenesis, and KBTBD11 preferentially interacts with Cullin3. The KBTBD11 and Cullin3 possibly cause ubiquitination of NFATc1. The ubiquitinated NFATc1 is probably degraded by the proteasome. Thus, it is likely that KBTBD11 negatively controls the protein levels of NFATc1 qualitatively and quantitatively at a post-translational level.

One of our important findings is that the KBTBD11 and Cullin3 possibly cause NFATc1 ubiquitination. Among the six major signaling pathways participating in osteoclast differentiation, only NFATc1 expression was correlated with osteoclast differentiation. KBTBD11 knockdown increased NFATc1 expression, and conversely, KBTBD11 overexpression decreased NFATc1 expression. Meanwhile, the phosphorylation levels of JNK, IκB, ERK, Akt, and p-38 were indistinguishable between control and KBTBD11-overexpressing RAW-D cells. These results also indicate that KBTBD11 and Cullin3 possibly regulate the quantitative regulation of NFATc1 expression.

Among seven Cullin proteins in mammalian cells, only Cullin3 associates with BTB-Kelch proteins. Accumulating evidence from biochemical and structural analyses has indicated that BTB-Kelch proteins directly bind to Cullin3 at N-terminal BTB domain^20,22,23^. Therefore, we can speculate that the N-terminal BTB domain of Cullin3 is important for the interaction with KBTBD11.

As a major function of BTB-Kelch proteins, some members are reported to participate in skeletal muscle development^21^. Mutations in human KLHL9^24^, KBTBD13^25^, KLHL40 (KBTBD5)^26–28^, and/or KLHL41 (KBTBD10)^29^, all of which interact with Cullin3, cause skeletal muscle disorders^21,23,30^. In the case of a lack of Kelch proteins, the absence of the ubiquitin ligase complex causes accumulation of abnormal proteins, resulting in skeletal muscle diseases^21^. Among these, the function of KLHL41 in myoblasts during muscle differentiation is reminiscent of that of KBTBD11 in RANKL-stimulated macrophages during osteoclast differentiation. Importantly, myoblasts undergo cell–cell fusion to form multinucleated cells, a process termed as myogenesis. KLHL41 expression is gradually increased in myoblasts during muscle differentiation^31^. Knockdown of KLHL41 causes enhanced differentiation at an early phase^32^. However, in the late phase of differentiation, the KLHL41 knockdown reduces the percentage of cells with more than five nuclei, thus pointing to successful inhibition of differentiation^32^. In an analogous fashion, the KBTBD11 knockdown enhanced osteoclastogenesis, while the KBTBD11 overexpression inhibited osteoclast differentiation in our study. Therefore, it is of interest to examine multinuclear mechanisms of BTB-Kelch proteins in osteoclastogenesis versus myogenesis.

Cell differentiation is strictly controlled by negative-feedback mechanisms to prevent over-stimulation. Since NFATc1 is an essential transcriptional factor for osteoclastogenesis^3,33^, it is regulated by many epigenetic mechanisms, such as de-acetylation, methylation, miRNA and ubiquitination^34^. Among these, it is likely that ubiquitination of NFATc1 is controlled by several E3 ubiquitin ligases. RING finger-type E3 ubiquitin ligases Cbl-b and c-Cbl, negatively regulate osteoclast formation^15^. During late-stage osteoclastogenesis, after M-CSF-mediated c-Src kinase activation, activated Cbl-b and c-Cbl cause NFATc1 ubiquitination, resulting in proteasomal degradation^15^. JMJD5, a Jumonji C domain-containing protein, also negatively controls osteoclast differentiation by promoting degradation of ubiquitinated NFATc1^35^. JMJD5 acts as a hydroxylase, thereby promoting the interaction of hydroxylated NFATc1 and E3 ubiquitin ligase complex, including von Hippel-Lindau tumor suppressor^35^. However, it is unclear how several ubiquitin ligases regulate NFATc1. It is possible to speculate that multiple negative regulatory mechanisms mediated by several ubiquitin ligases are required for the maintenance of NFATc1 for bone homeostasis, since NFATc1 has auto-amplification mechanisms.

In conclusion, this study shows that KBTBD11 negatively regulates osteoclast differentiation by interaction with Cullin3 via NFATc1 degradation.

## Methods

### Reagents

Recombinant RANKL was prepared as described previously^36^. α-Minimum essential medium (α-MEM) was purchased from WAKO (Osaka, Japan). Fetal bovine serum was obtained from Sigma-Aldrich (St. Louis, MO, USA). Rabbit polyclonal anti-GFP (Cat. No. 598) was from MBL (Nagoya, Japan). Rabbit monoclonal anti-phospho-p38 (Cat. No. 4511S, Thr180/Tyr182), rabbit monoclonal anti-phospho-IκBα (Cat. No. 2859S, Ser32), rabbit polyclonal anti-phospho-JNK (Cat. No. 9251S, Thr183/Tyr185), rabbit monoclonal anti-phospho-Akt (protein kinase B) (Cat. No. 4060S, Ser473), rabbit polyclonal anti-phospho-ERK1/2 (Cat. No. 9101S, Thr202/Tyr204), rabbit monoclonal anti-glyceraldehyde-3-phosphate dehydrogenase (GAPDH) (Cat. No. 2118S) antibodies were from Cell Signaling Technology (Danvers, MA, USA). Anti-Cullin3 antibody (CUL3-9, C-terminal #ab214765) and anti-KBTBD11 antibody (#ab151125) were purchased from Abcam PLC (Cambridge, UK). Mouse monoclonal anti-NFATc1 (nuclear factor of activated T-cells, cytoplasmic 1) (Cat. No. sc-7294) were from Santa Cruz Biotechnology (Santa Cruz, CA, USA). Mouse monoclonal anti-Src (proto-oncogene tyrosine-protein kinase) (Cat. No. 05-184) was purchased from Upstate Biotechnology (Lake Placid, NY, USA). Anti-CTSK antibody was prepared as previously described^37^. All other reagents, including phenylmethylsulfonyl fluoride (PMSF) and the protease inhibitor cocktail, were obtained from Sigma-Aldrich. MLN4924 (Cat. No. CEM-CS-0348) was purchased from Chemscene LLC (Monmouth Junction, NJ, USA).

### Gene knockdown by siRNA

A synthetic siRNA oligonucleotides specific for *KBTBD11* (#1siRNA: 5′-UCCUGAGACAUCUUUGCCCTT-3′; #2siRNA: 5′-AGGUGUACAGCACAUUGG -3′; #3siRNA: 5′-AGUGGUCGCUCAUGAAGC-3′) were designed and synthesized by Invitrogen (Carlsbad, CA, USA). RAW-D cells were transfected with the siRNA oligonucleotide (20 nM/transfection), using Lipofectamine RNAiMAX (Invitrogen). After 48 h of transfection, the cells were allowed to differentiate into osteoclasts in the presence of RANKL for 4 or 6 days. Stealth siRNA Negative Control Duplexes (Invitrogen) served as a negative control.

### Retrovirus construction and overexpression of KBTBD11

Retrovirus construction and overexpression experiments were performed according to previously described methods^38^. Briefly, the full length cDNA of mouse KBTBD11 ORF Clone (MG217626) was purchased from OriGene. The cDNAs were amplified by PCR using PrimeSTAR GXL DNA polymerase (Takara, Tokyo). To express the KBTBD11-GFP fusion protein, the amplified fragments were fused with a linearized pMSCVpuro-GFP using In-Fusion cloning kit (Clontech, Mountain View, CA, USA). A control vector was composed of only GFP cDNA. All vectors placed GFP at its N terminus. Vectors were transfected into HEK293T cells using the Lipofectamine 2000 kit (Life Technologies, Gaitherburg, MD, USA). After incubation at 37 °C and 5% CO_2_ for 48 h, the virus-containing supernatants were collected and used to infect RAW-D cells. KBTBD11 overexpressing cells were selected on puromycin (3 μg/mL) in α-MEM and every 3 days the medium was refreshed. About 2 weeks later, several cell clones were obtained.

### Immunoprecipitation

Cells were washed twice with phosphate-buffered saline (PBS). Then, the cells were lysed in a lysis buffer (PBS containing 0.1% Triton X100, 10 mM N-ethylmaleimide, and proteinase inhibitors). After centrifugation at 25,000 × *g* for 10 min at 4 °C, the supernatants were immunoprecipitated with anti-Cullin3 anti-body that was immobilized on resin using the Co-immunoprecipitation Kit (Pierce, Thermo Scientific). For immunoprecipitation experiments with ubiquitinated NFATc1, the cells were lysed in the lysis buffer containing 10 mM N-ethylmaleimide. The cell lysates were quenched by 0.1% cysteine, and incubated with anti-NFATc1 antibody for 1 hr followed by overnight incubation with protein G–Sepharose at 4 °C. After addition of protein G–Sepharose, the beads were washed four times with the lysis buffer, and then washed once with PBS. The sedimented beads were resuspended in Laemmli’s sample buffer containing 100 mM DTT, were boiled and subjected to western blot analysis.

### Western blot analysis

Western blot analysis was performed according to previously described methods^38^. Briefly, after washing, cells were lysed in a cell lysis buffer [50 mM Tris-HCl (pH 8.0), 1% Nonidet P-40, 0.5% sodium deoxycholate, 0.1% sodium dodecyl sulfate (SDS), 150 mM NaCl, 1 mM PMSF, and proteinase inhibitor cocktail]. The same protein amounts (5 µg) were subjected to SDS-polyacrylamide gel electrophoresis (PAGE), followed by transfer onto a polyvinylidene difluoride membrane. The blots were blocked with 5% milk in Tris-buffered saline for 1 h at 25 °C, incubated with various primary antibodies overnight at 4 °C, washed, incubated with horseradish peroxidase-conjugated secondary antibodies, and finally detected with ECL-Prime (GE Healthcare Life Sciences, Tokyo, Japan). The immunoreactive bands were analyzed using a LAS-4000mini (Fujifilm, Tokyo, Japan).

### Quantitative PCR analysis

Total RNA was extracted using the TRIzol Reagent (Invitrogen). Reverse transcription was performed using an oligo(dT)15 primer (Promega, Madison, WI, USA) and ReverTra Ace (Toyobo, Osaka, Japan). Quantitative RT-PCR was performed using an MX3005P QPCR system (Agilent Technologies, La Jolla, CA, USA). The cDNA was amplified using Brilliant III Ultra-Fast SYBR QPCR Master Mix (Agilent). The following primer sets were used (5′ to 3′): KBTBD11, forward: GTT TCG TGC CTT CTC AGG AC, and reverse: GCT TCT CAG GCA GGT TGA GT; NFATc1, forward: TCATCCTGTCCAACACCAAA and reverse: TCACCCTGGTGTTCTTCCTC;

OC-STAMP, forward: TGGGCCTCCATATGACCTCGAGTAG and reverse: TCAAAGGCTTGTAAATTGGAGGAGT; DC-STAMP, forward: CTAGCTGGCTGGACTTCATCC and reverse: TCATGCTGTCTAGGAGACCTC;

SRC (Src), forward: AGAGTGCTGAGCGACCTGTGT and reverse: GCAGAGATGCTGCCTT-GGTT; CTSK, forward, CAGCTTCCCCAAGATGTGAT; reverse: AGCACCAACGAGAGGAGAAA; CALCR (calcitonin receptor), forward: CGCATCCGCTTGAATGTG and reverse: TCTGTCTTTCCCCAG-GAAATGA; MMP9, forward, TATTTTTGTGTGGCGTCTGAGAA and reverse: GAGGTGGTTTAGCCGGTGAA; GAPDH, forward: ACCACAGTCCATGCCATCAC; reverse: TCCACCACCCTGTTGCTGTA.

### TRAP staining

TRAP staining was performed by the method described previously^38^. Briefly, after fixation with 4% paraformaldehyde, cells were incubated with 0.01% naphthol AS-MX phosphate (Sigma-Aldrich) and 0.05% fast red violet LB salt (Sigma-Aldrich, Tokyo, Japan) in the presence of 50 mM sodium tartrate and 90 mM sodium acetate (pH 5.0) for TRAP activity. TRAP-positive red cells with three or more nuclei were considered as mature osteoclasts.

### Bone resorption assay

Bone resorption assay was performed by the method described previously^38^. Briefly, RAW-D cells were seeded onto Osteo Assay Stripwell Plates coated with thin calcium phosphate films (Corning, New York, USA), and incubated with RANKL (500 ng/mL) for 1 day. After that, the cells were incubated with siRNA (control or KBTBD11-specific), and then cultured for 6 days until multinucleated osteoclasts were formed in the presence of 500 ng/mL RANKL. After incubation, osteoclasts from RAW-D cells were dissolved in 5% sodium hypochlorite. The ratios of the resorbed areas to the total areas were calculated using the Image J software (http://rsbweb.nih.gov/ij/) as described previously.

### Statistical analysis

All values were expressed as means ± standard deviation (SD) of three independent experiments. Data were analyzed by the Tukey-Kramer method when analysis of variance (ANOVA) indicated a significant difference between concentrations (*P < 0.05 or **P < 0.01).

## Supplementary information

Supplement information
