# Supplementary material for: KBTBD11, a novel BTB-Kelch protein, is a negative regulator of osteoclastogenesis through controlling Cullin3-mediated ubiquitination of NFATc1
Source: Sci Rep. 2019 Mar 5;9:3523. doi: 10.1038/s41598-019-40240-2 (PMC6401029; doi:10.1038/s41598-019-40240-2)
Supplement: Supplementary file 1 — Supplement information [file 41598_2019_40240_MOESM1_ESM.pdf]

Supplementary Information

KBTBD11, a novel BTB-Kelch protein, is a negative regulator of osteoclastogenesis through controlling Cullin3-mediated ubiquitination of NFATc1

Shun Narahara[1, 2], Eiko Sakai[1], Tomoko Kadowaki[3], Yu Yamaguchi[1], Haruna Narahara[1], Kuniaki Okamoto[1, 4], Izumi Asahina[2], and Takayuki Tsukuba[1]*

[1]Department of Dental Pharmacology, Graduate School of Biomedical Sciences, Nagasaki University, Nagasaki 852-8588, Japan.

[2]Department of Regenerative Oral Surgery, Graduate School of Biomedical Sciences, Nagasaki University, Nagasaki 852-8588, Japan.

[3]Department of Frontier Life Science, Graduate School of Biomedical Sciences, Nagasaki University, Nagasaki 852-8588, Japan.

[4]Department of Dental Pharmacology, Okayama University Graduate School of Medicine, Dentistry and Pharmaceutical Sciences, Okayama, 700-8525, Japan.

Contents

Supplementary Figures

Figure S1: Comparison of mRNA levels of various osteoclast marker genes in control and KBTBD11-knockdown osteoclasts…………………………………………………………………………………..….3

Figure S2: Comparison of mRNA levels of various osteoclast marker genes in control and KBTBD11-overexpressing osteoclasts.……………………………………………………………………………...…4

Figure S3: Bone resorbing activities of control and KBTBD11-overexpressing osteoclasts.……………………………………………………………………………...…5

Figure S4: Nuclear translocation of NFATc1 in control and KBTBD11-overexpressing osteoclasts.……………………………………………………………………………..……6

Figure S5~S13: Original gel images of immunoblot analysis.……………. ……………………7

Figure legends: ………………………………………………………………..………….16

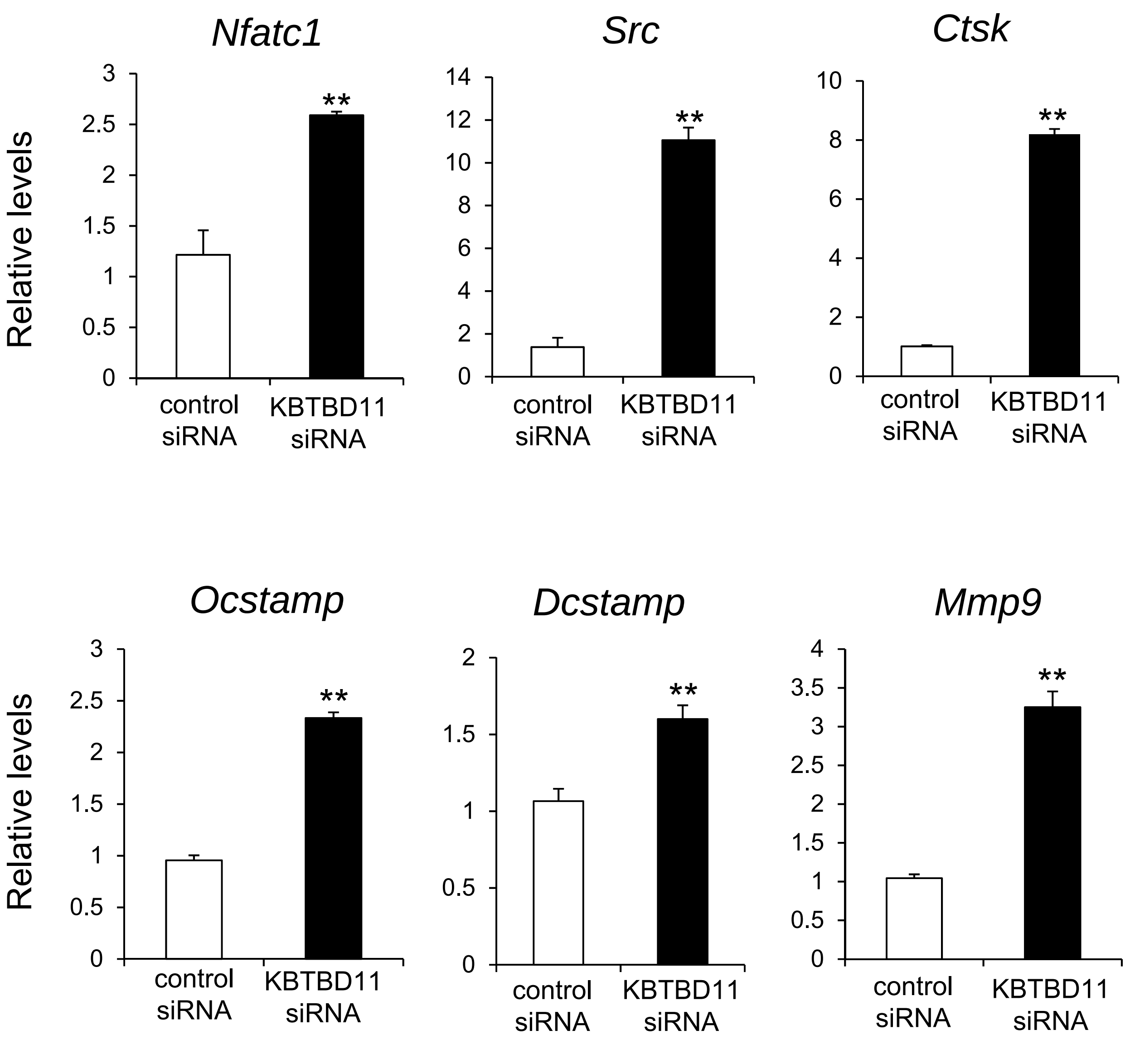

Supplement Figure S1

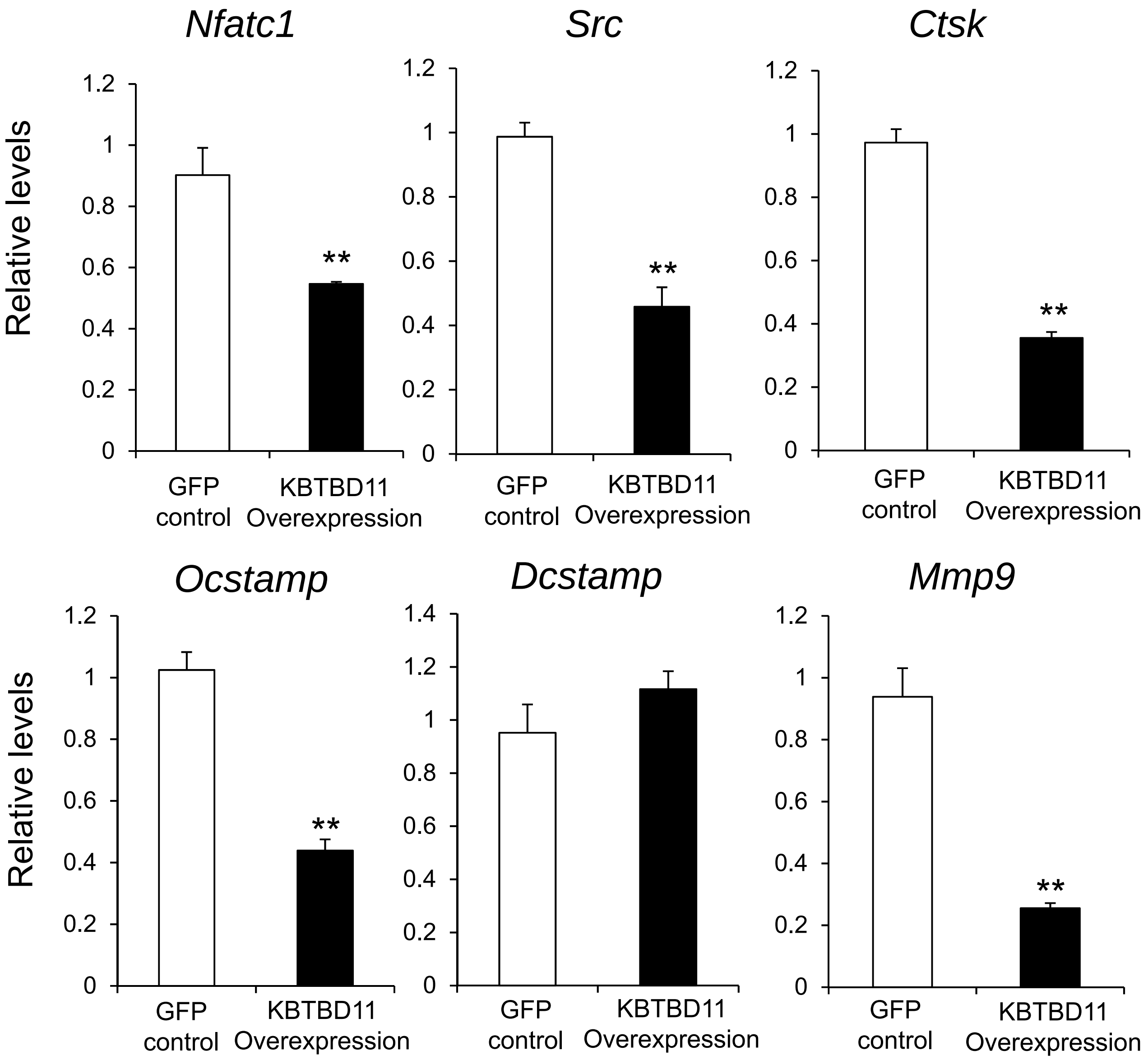

Supplement Figure S2

a

GFP control

KBTBD11 overexpression

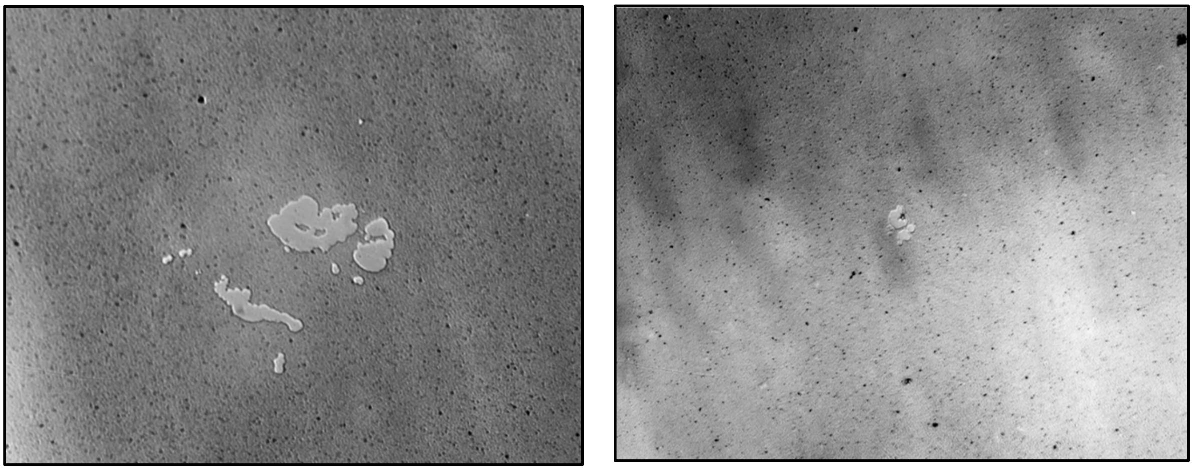

b

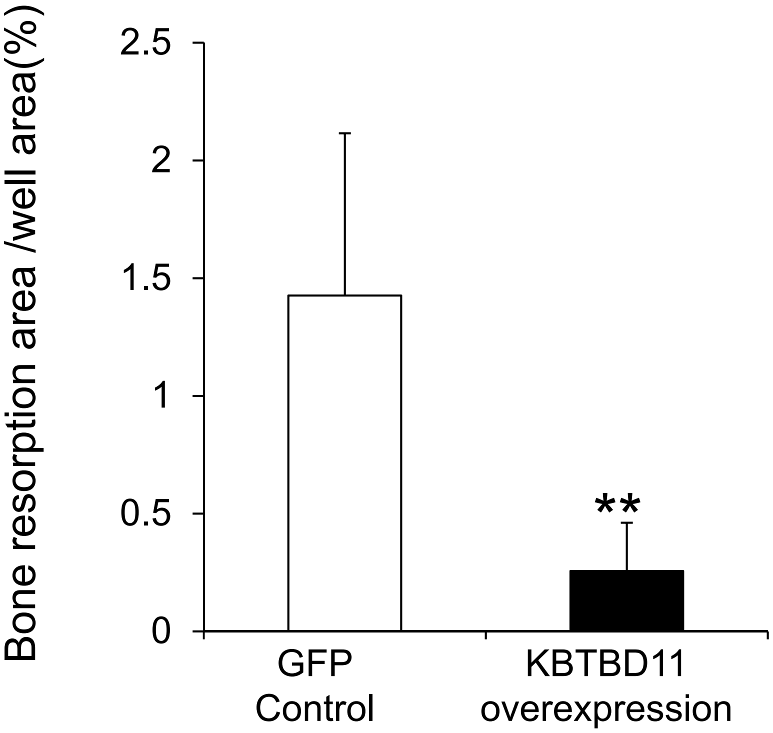

Supplement Figure S3

a

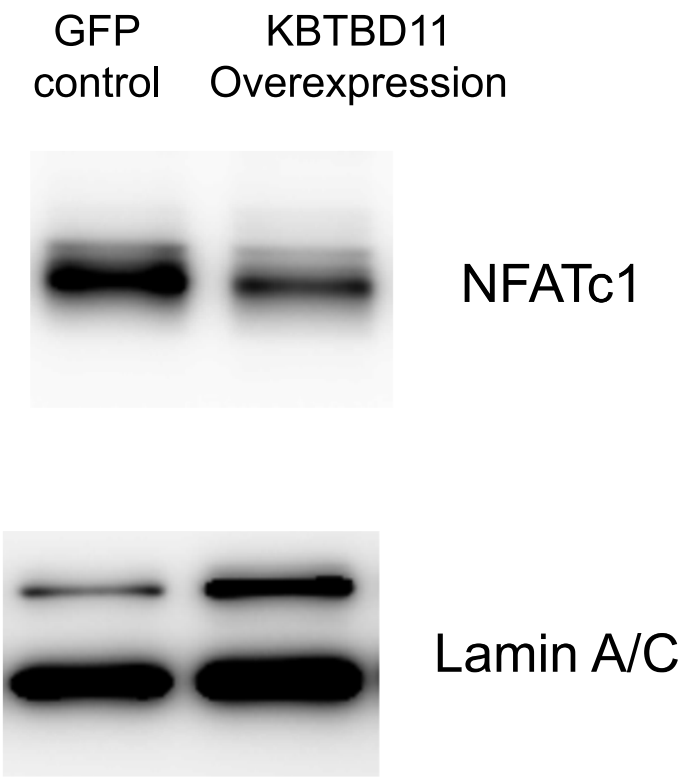

b

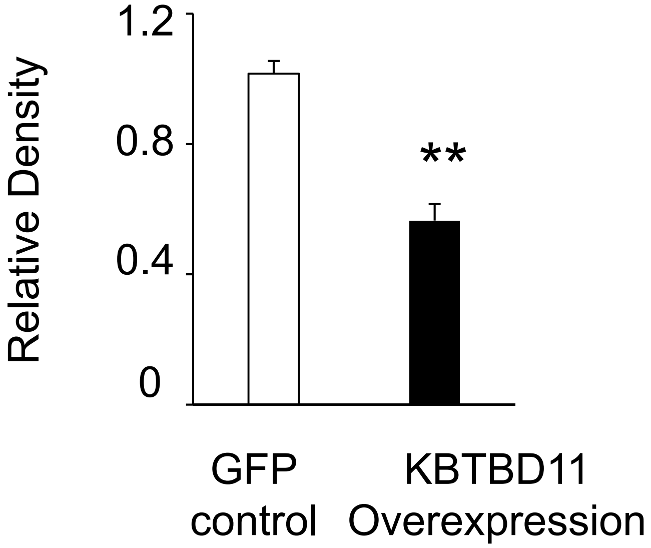

Supplement Figure S4

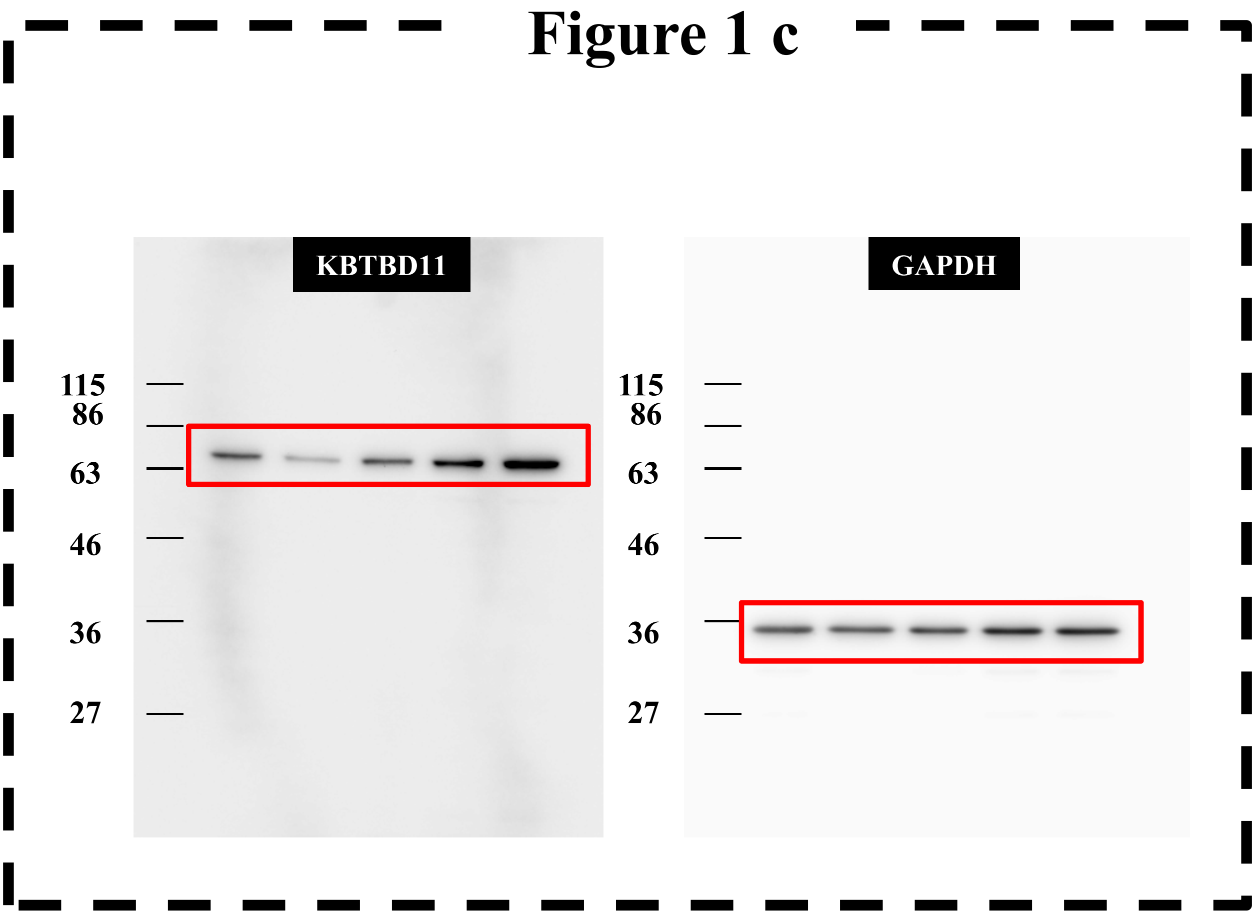

Supplement Figure S5

Figure 4 a

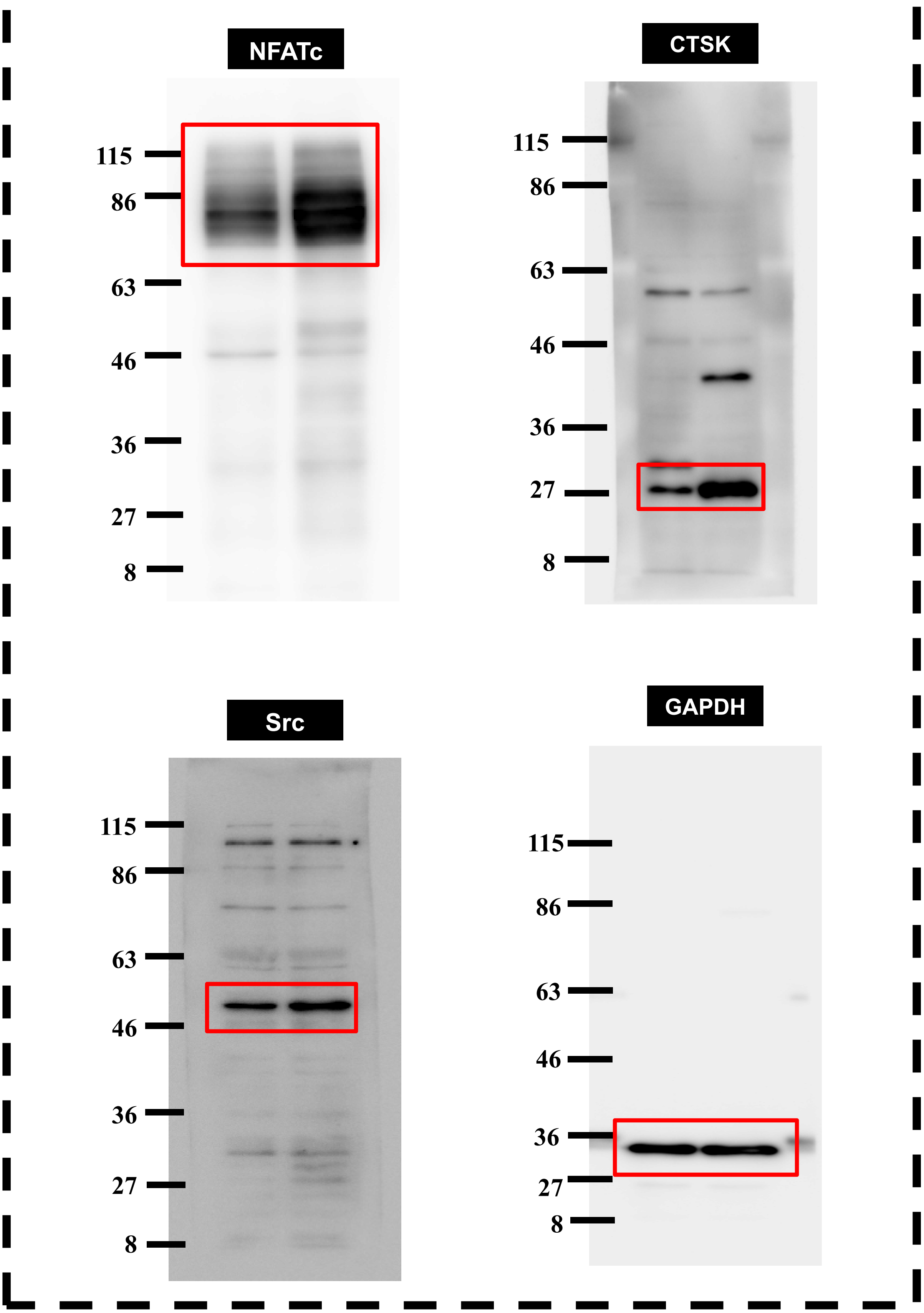

Supplement Figure S6

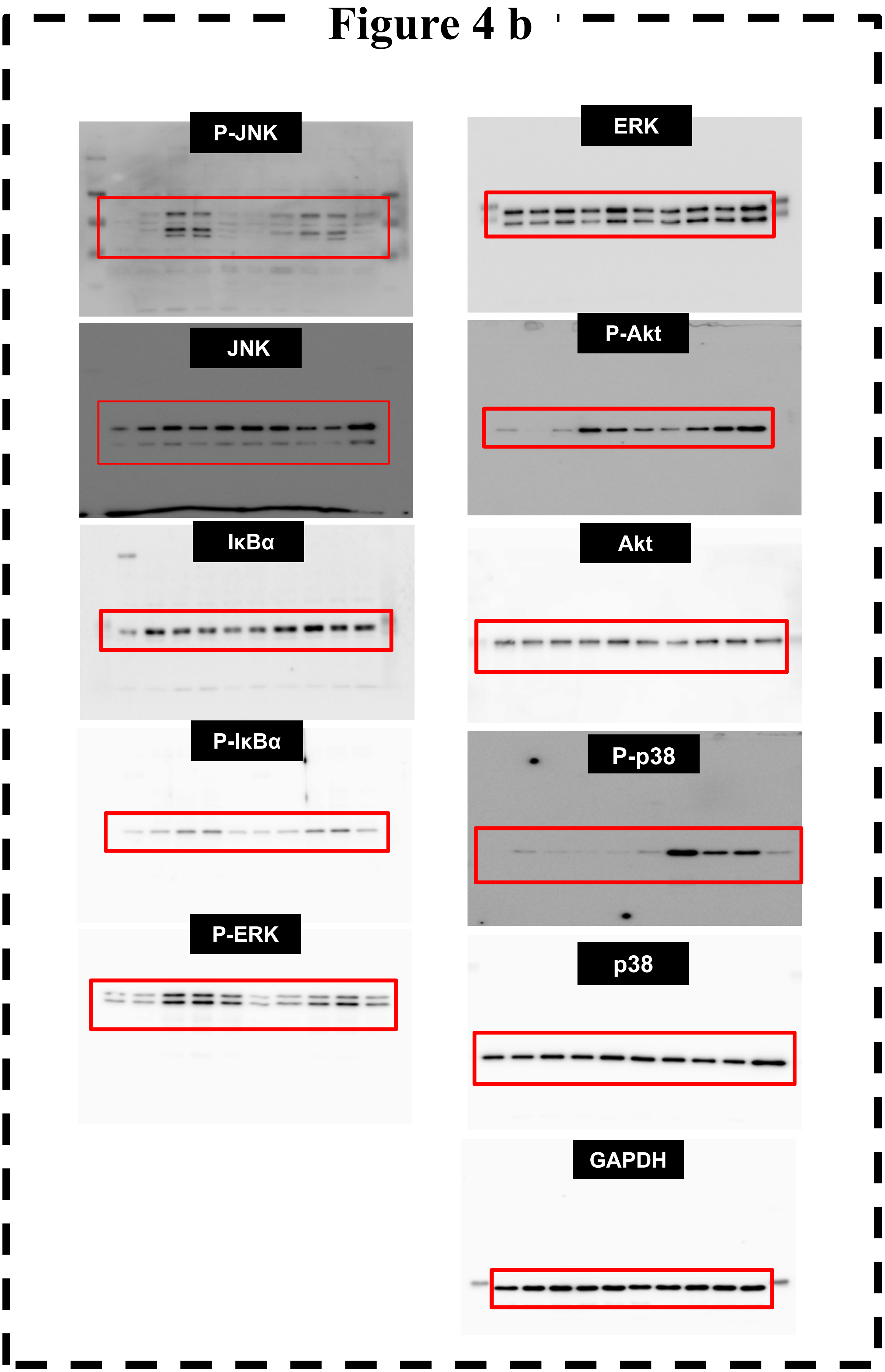

Supplement Figure S7

Figure 6 a

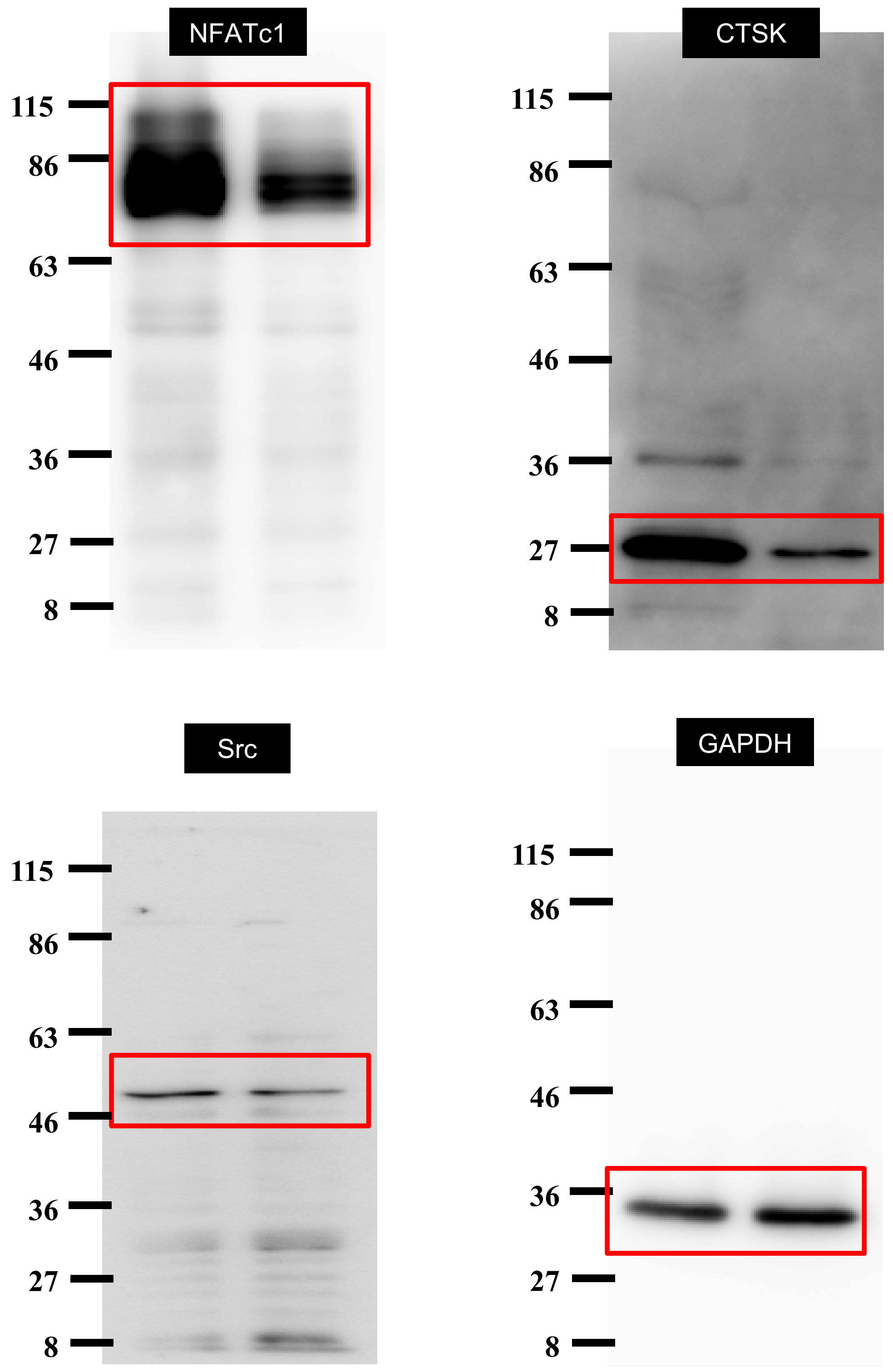

Supplement Figure S8

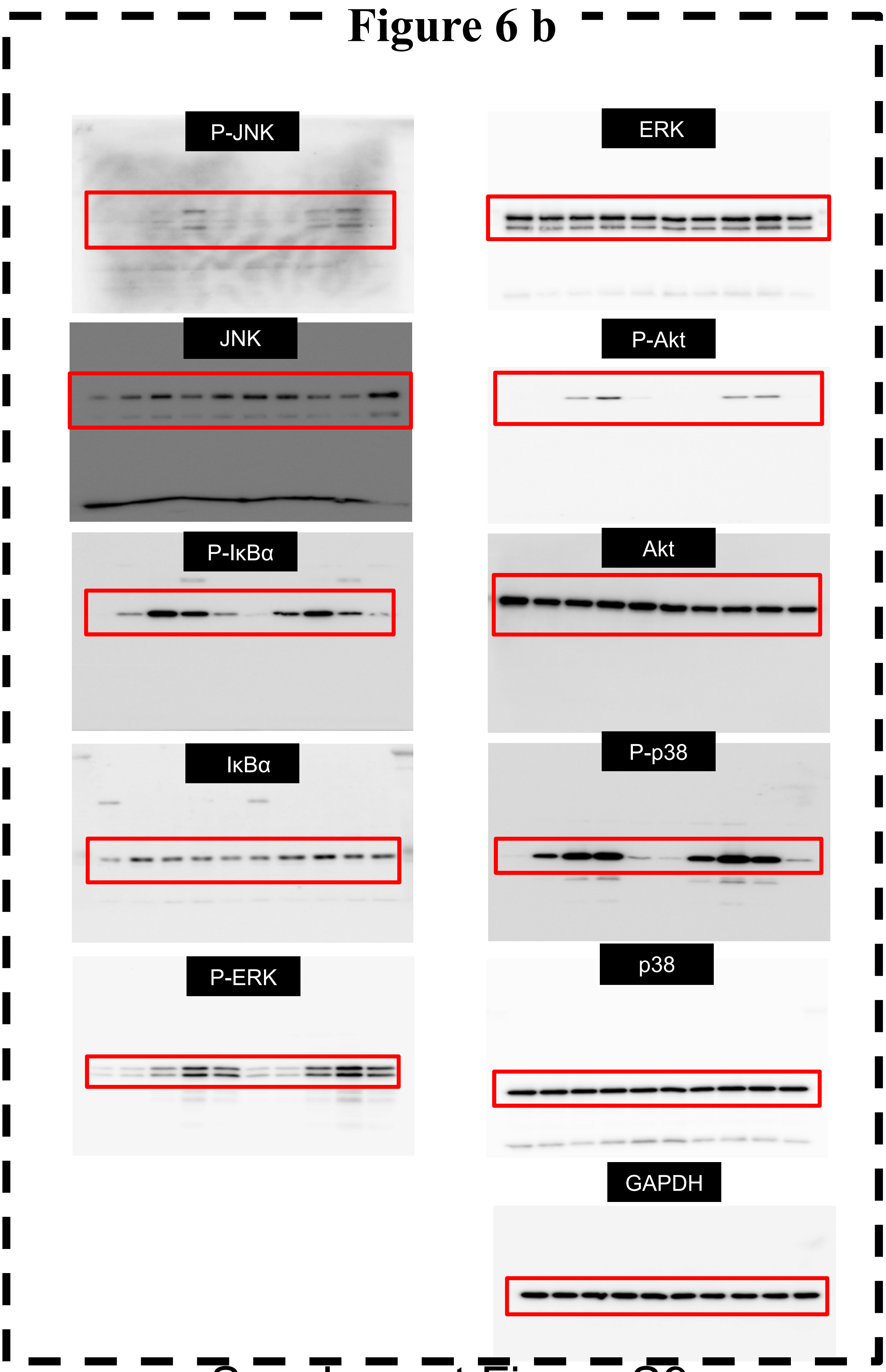

Supplement Figure S9

Figure 7

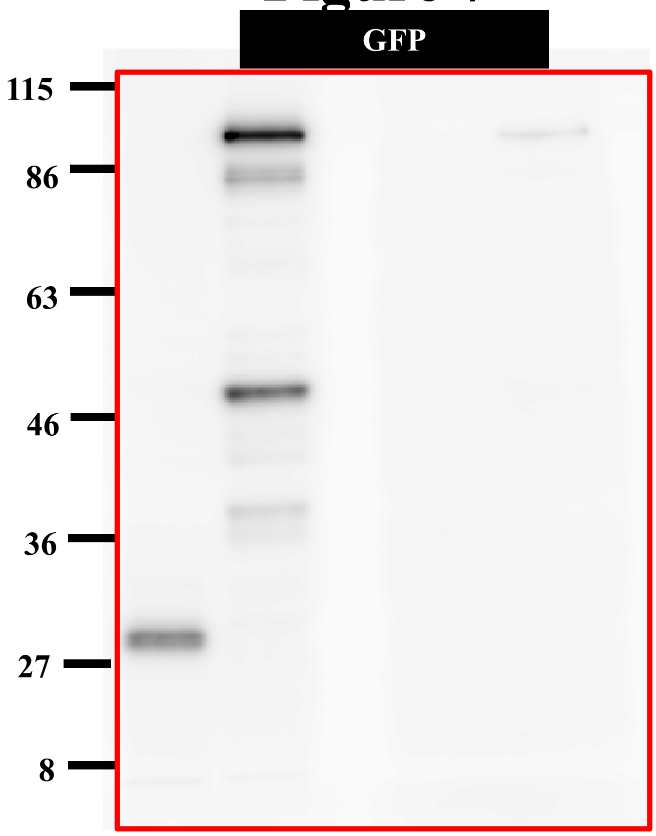

Supplement Figure S10

Figure 8

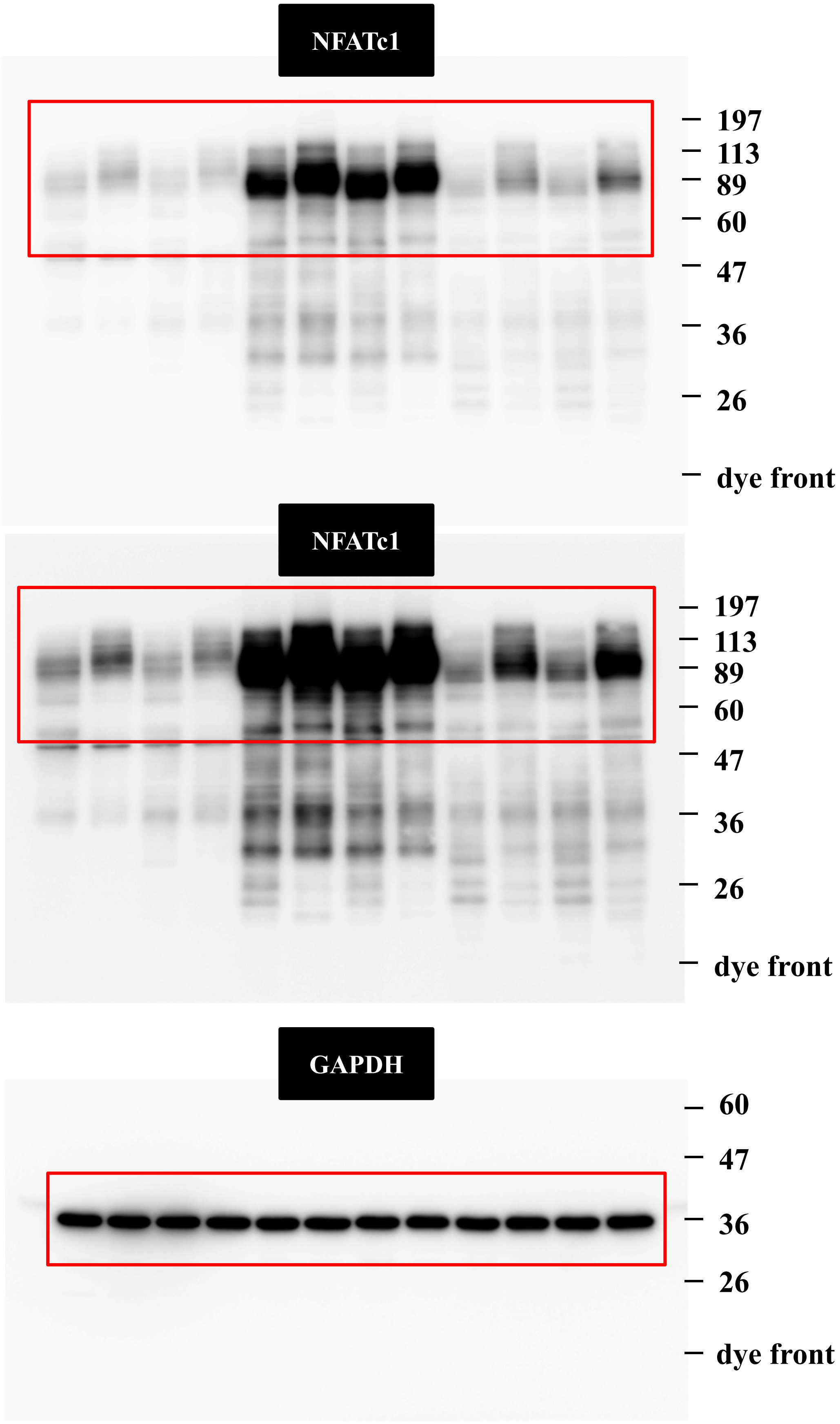

Supplement Figure S11

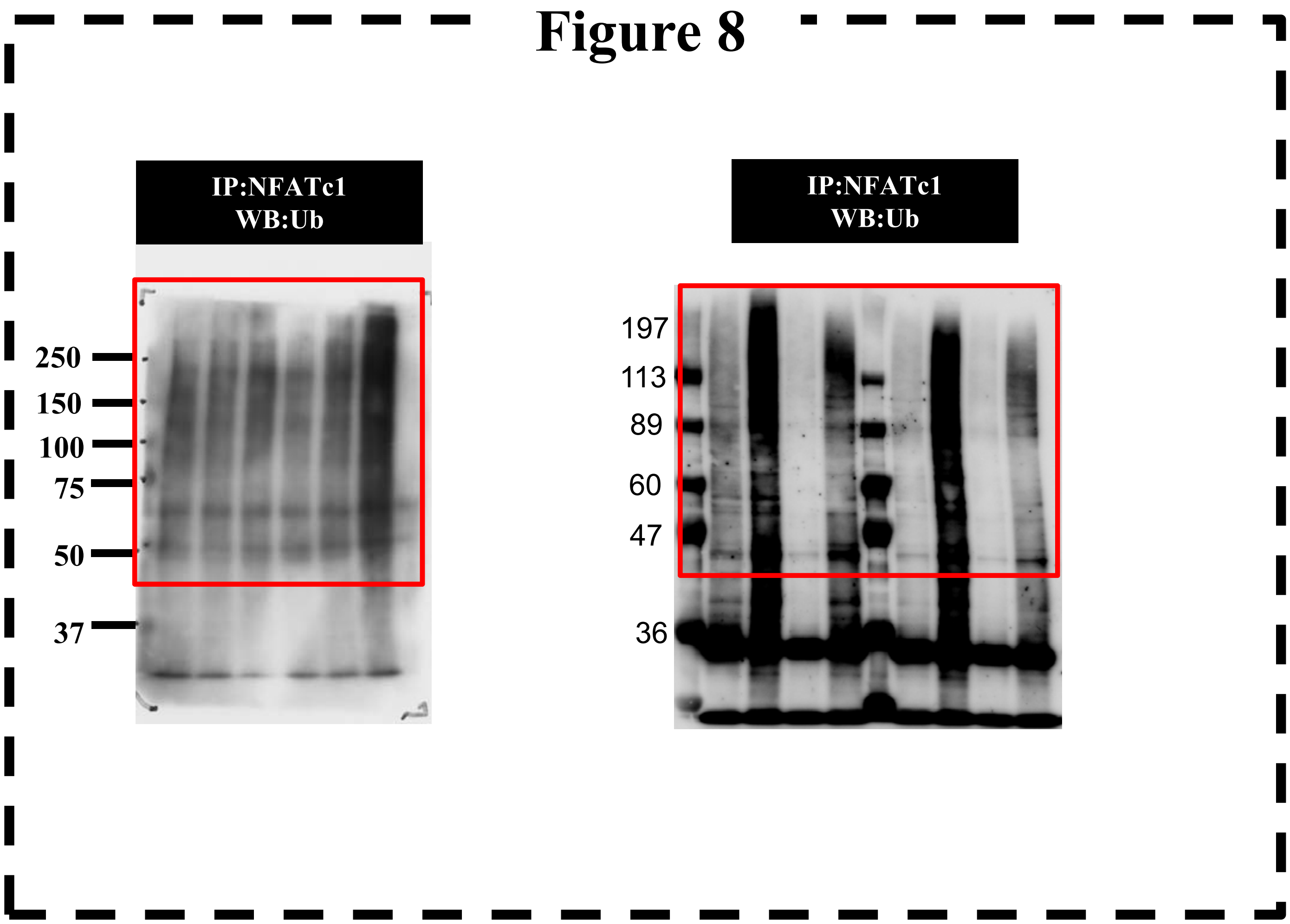

Supplement Figure S12

Supplement Figure 4

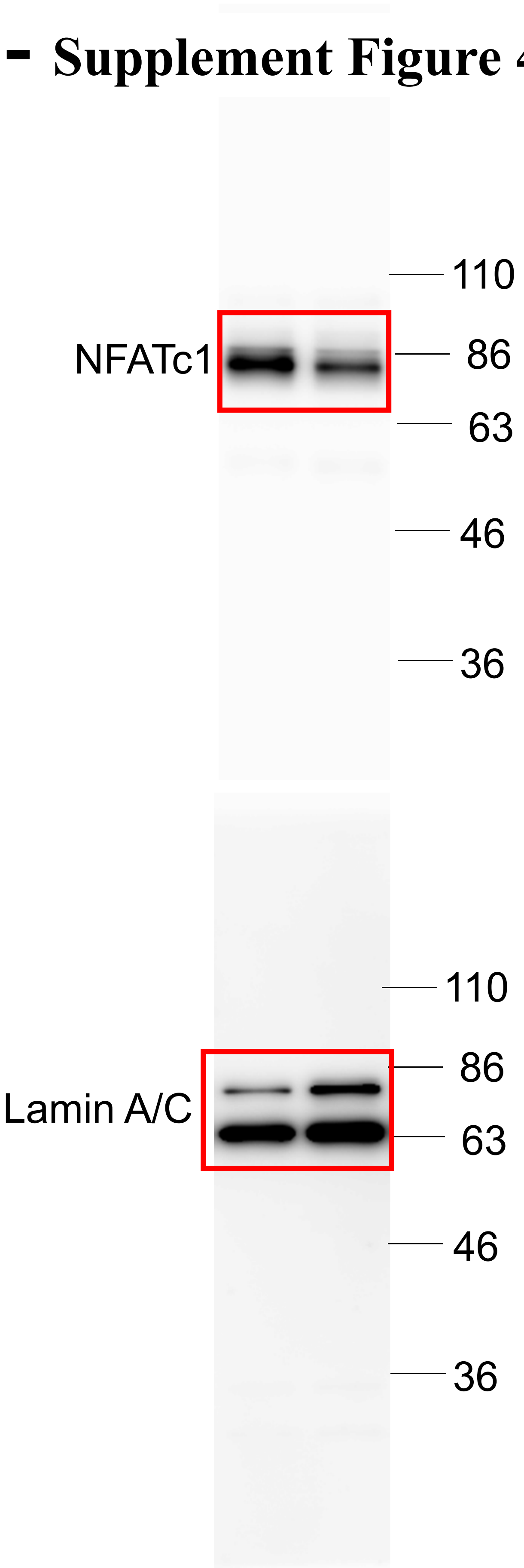

Supplement Figure S13

Supplement Figure S1. Comparison of mRNA levels of various osteoclast marker genes in control and KBTBD11-knockdown osteoclasts. Control and KBTBD11-knockdown RAW-D cells were cultured with RANKL (100 ng/mL) for 3 days. After isolation of mRNA, RT-PCR was performed. **P < 0.01, compared with control cells.

Supplement Figure S2. Comparison of mRNA levels of various osteoclast marker genes in control and KBTBD11-overexpressing osteoclasts. Control and KBTBD11-overexpressing RAW-D cells were cultured with RANKL (100 ng/mL) for 3 days. After isolation of mRNA in these cells, RT-PCR was performed. **P < 0.01 compared with the control cells.

Supplement Figure S3. Bone resorbing activities of control and KBTBD11-overexpressing osteoclasts.

(a) Control and KBTBD11-overexpressing RAW-D cells were seeded on Osteo Assay Stripwell Plates with RANKL (500 ng/mL) and incubated for 7 days. Photographs of the bone-resorbing activity of each osteoclast. (b) The resorption area was determined using Image J software. **P < 0.01 compared with control or transfected cells.

Supplement Figure S4: Nuclear translocation of NFATc1 in control and KBTBD11-overexpressing osteoclasts.

(a) Cells were cultured with RANKL (100 ng/mL) for 48h. Nuclear fractions were prepared using a nuclear extraction kit (Active Motif, Carlsbad, CA, USA) according to the manufacturer's instruction. Nuclear extracts were analyzed by western blotting with indicated antibodies. Lamin A/C was used as a loading control. Western blots were quantified using image J software. Numbers below each band represent the relative intensity normalized to the corresponding values of lamin A/C.

(b) Densitometric analysis for the quantification of each protein in the cell lysate of both cell types as shown in (a). The relative levels were defined as the chemiluminescence intensity per mm^2 measured by LAS4000-mini. The data are presented as the mean ± S.D. of values from 3 independent experiments. $^{**}P < 0.01$ for the indicated comparison.
